# Highly selective fluorescent chemosensor for detection of Fe^3+^ based on Fe_3_O_4_@ZnO

**DOI:** 10.1038/srep23558

**Published:** 2016-03-22

**Authors:** Jingshuai Li, Qi Wang, Zhankui Guo, Hongmin Ma, Yong Zhang, Bing Wang, Du Bin, Qin Wei

**Affiliations:** 1 Key Laboratory of Chemical Sensing & Analysis in Universities of Shandong, School of Chemistry and Chemical Engineering, University of Jinan, Jinan 250022, P.R. China; 2 School of Material Science and Engineering, University of Jinan, Jinan 250022, P.R. China

## Abstract

The combination of fluorescent nanoparticles and specific molecular probes appears to be a promising strategy for developing fluorescent nanoprobes. In this work, L-cysteine (L-Cys) capped Fe_3_O_4_@ZnO core-shell nanoparticles were synthesized for the highly selective detection of Fe^3+^. The proposed nanoprobe shows excellent fluorescent property and high selectivity for Fe^3+^ due to the binding affinity of L-Cys with Fe^3+^. The binding of Fe^3+^ to the nanoprobe induces an apparent decrease of the fluorescence. Thus a highly selective fluorescent chemosensor for Fe^3+^ was proposed based on Fe_3_O_4_@ZnO nanoprobe. The magnetism of the nanoprobe enables the facile separation of bound Fe^3+^ from the sample solution with an external magnetic field, which effectively reduces the interference of matrix. The detection limit was 3 nmol L^−1^ with a rapid response time of less than 1 min. The proposed method was applied to detect Fe^3+^ in both serum and wastewater samples with acceptable performance. All above features indicated that the proposed fluorescent probe as sensing platform held great potential in applications of biological and analytical field.

The development of highly sensitive fluorescent probes for the selective detection of heavy metal ions and transition metals has been inspiring the scientific community in the past few years as a result of concern for human health and environmental safety^1^,^2^,^3^,^4^,^5^. Among them, iron ion is not only one of the heavy metal ions but also one of the most essential trace elements in human body. The maximum level of Fe^3+^ permitted in drinking water is 5.4 μmol L^−1^ by the U.S. Environmental Protection Agency^6^. And it presents in many enzymes and proteins and acts as cofactor for many cellular metabolism reactions^7^. Many physiological processes could not miss the participation of iron, such as oxygen transportation, oxygen metabolism, transcriptional regulation and electron transfer^8^,^9^. In particular, iron ion in blood can promote the formation of red blood proteins. And the lack of iron can lead to anemia^10^. However, excess iron contents may also impair biological systems, because its redox-active form catalyzes the generation of highly reactive oxygen species^11^, which involves in kinds of diseases including Parkinson’s syndrome, Alzheimer’s disease and cancer^12^,^13^,^14^. Therefore, the assay of iron levels has been an active issue in environmental and biomedical analysis.

By now, many methods have been raised for the detection of Fe^3+^ such as atomic absorption spectroscopy^15^, colorimetric analysis^16^, mass spectrometry^17^ electrochemical^18^,^19^ and fluorescence spectroscopic analysis^20^,^21^,^22^,^23^,^24^,^25^,^26^. Among these methods, fluorimetric assay is a favorable method due to its ease of operation, high sensitivity and efficiency. Therefore, the design of fluorescent probes for detecting Fe^3+^ has attracted increasing attentions. The successful Fe^3+^ fluorescent probes mainly limited to organic fluorescent molecular^20^,^21^,^22^,^23^,^24^,^25^,^26^, quantum dots^27^,^28^ and their complexes^29^,^30^. However, organic dyes involved in complicated synthesis route and poor photostability. Quantum dots such as CdSe and CdTe are toxic to biological systems^31^. Therefore, designing appropriate nanoprobes toward synthesis facile, photostable and environmental friendly orientation for detecting Fe^3+^ is still a worthwhile and challenging undertaking.

ZnO nanoparticles are currently intensively studied as photocatalysts, sensors and phosphors. It was reported that ZnO nanoparticles were able to penetrate living cells and were generally nontoxic^32^. Therefore, ZnO nanoparticles are ideal candidates as replacement for Cd-based fluorescent labels since they are nontoxic, less expensive and chemically stable in air. Magnetite Fe_3_O_4_ as commercial nanomaterial has strong magnetism, magnetic manipulability and good biocompatibility. Also it has widespread applications in magnetic bioseparation^33^, drug delivery^34^ and magnetic resonance imaging^35^. In this work, we develop an L-cysteine capped magnetic Fe_3_O_4_@ZnO nanosensor (Fe_3_O_4_@ZnO@L-Cys) for detection and removal of Fe^3+^ (Fig. 1). The results showed that Fe_3_O_4_@ZnO@L-Cys quantificationally detected Fe^3+^ with high sensitivity and selectivity under a pH range (pH 4.98–7.39) and could remove Fe^3+^ from the water sample. Moreover, the fabricated magnetic fluorescent probe could be removed by external magnetic field, and the potential secondary pollution was avoided.

## Experimental

### Regents and apparatus

Fe_3_O_4_ nanoparticles were purchased from Aladdin Chemical Co., Ltd. Zinc acetate (Zn(Ac)_2_) was purchased from Tianjin Hongyan Chemical Reagent Factory. Triethanolamine was purchased from Guangcheng Chemical Reagent Co., Ltd. (Tianjin). L-Cys was purchased from Yunxiang Chemical Industry Co., Ltd. Absolute ethyl alcohol was purchased from Fuyu Chemical Reagent Factory. All other reagents used in this study were analytical grade, and ultrapure water was used in the preparation of all solutions.

Transmission electron microscope (TEM) images were obtained from a Tecnai G220 TEM (FEI Company, USA). Energy Dispersive X-Ray Spectroscopy (EDS) was recorded by JEOL JSM-6700 F microscope (Japan). FT-IR spectra were collected using a FT-IR-410 infrared spectrometer (JASCO, Japan). Ultraviolet absorption spectra were obtained from a Lambda35 UV-Vis spectrophotometer (PerkinElmer, America). Fluorescence spectra were obtained from a LS-55 fluorescence spectrophotometer (PerkinElmer, America).

### Preparation of Fe_3_O_4_@ZnO@L-Cys

The Fe_3_O_4_@ZnO was prepared according to the published procedure^36^. 60 mg of L-Cys was dispersed into 20 mL of ethanol solution by sonication for 20 min in 100 mL conical flask. Then, 10 mg of Fe_3_O_4_@ZnO was added into the conical flask. The flask was wrapped with aluminum foil and vigorous stirring for 6 h. The L-Cys was linked on the surface of Fe_3_O_4_@ZnO by thiol groups of L-Cys^37^. The product was magnetically collected and washed with ultrapure water and ethanol for four times, respectively. The sample of Fe_3_O_4_@ZnO@L-Cys was re-dispersed into 50 mL ethanol solution (Fe_3_O_4_@ZnO@L-Cys stocking solution).

### Effect of pH values and ionic strength

The effect of pH values was studied as follows: 300 μL of Fe_3_O_4_@ZnO@L-Cys stocking solution was suspended in 2.7 mL of phosphate buffered saline (PBS) (20 mmol L^−1^) aqueous solution in colorimetric cylinder at different pH values (4.98, 5.83, 6.30, 7.02, 7.39, 7.95 and 8.35, respectively). The suspension was laid aside for 5 min and the emission spectra of the suspension were measured. Then, 200 μL of Fe^3+^ (2 mmol L^−1^) was added respectively. The suspension was laid aside for another 5 min and the emission spectra of the suspension were measured.

To test the influence of ionic strength on the fluorescence of Fe_3_O_4_@ZnO@L-Cys before and after the addition of Fe^3+^, a series of Fe_3_O_4_@ZnO@L-Cys solutions containing different concentrations of NaCl (0.33, 0.99, 1.98, 2.97, 3.96 and 4.95 mmol L^−1^) was prepared and the emission spectra was then measured.

### Time course of the Fe_3_O_4_@ZnO@L-Cys toward Fe^3+^

The response time of Fe_3_O_4_@ZnO@L-Cys toward Fe^3+^ was carried out as follows: 300 μL of Fe_3_O_4_@ZnO@L-Cys stocking solution was suspended in 2.7 mL of PBS (20 mmol L^−1^, pH 7.02) aqueous solution. Then the fluorescence intensity was tested. Subsequently, 300 μL of Fe^3+^ was added into the above solution. The fluorescence intensity was tested again every other 30 s for 10 min.

### Determination of the standard solution of Fe^3+^

The quantification of Fe^3+^ adsorbed by Fe_3_O_4_@ZnO@L-Cys was carried out as follows: 300 μL of Fe_3_O_4_@ZnO@L-Cys stocking solution was added in 2.7 mL of PBS (20 mmol L^−1^, pH 7.02) aqueous solution. Then the emission spectra of the Fe_3_O_4_@ZnO@L-Cys suspension with different concentrations of Fe^3+^ (0, 0.01, 0.1, 5, 50, 100, 133, 200, 300, 400 μmol L^−1^) were measured respectively.

### Selectivity and stability of Fe_3_O_4_@ZnO@L-Cys

In addition, the selectivity of Fe_3_O_4_@ZnO@L-Cys toward Fe^3+^ over other metal ions was investigated. The selective and sensitive adsorption experiments were also conducted at PBS (20 mmol L^−1^, pH 7.02) with Fe^3+^ (50 μmol L^−1^) and other metal ions (Pb^2+^, Cr^3+^, Cd^2+^, Mg^2+^, Mn^2+^, Cu^2+^, Co^2+^ and Al^3+^, 200 μmol L^−1^) in the solutions. The emission spectra of the Fe_3_O_4_@ZnO@L-Cys suspension were measured respectively.

To evaluate the stability of Fe_3_O_4_@ZnO@L-Cys, the emission spectra was measured every other 10 d.

### Removal of Fe^3+^ from the standard solution

The removal ability of Fe_3_O_4_@ZnO@L-Cys from standard solution was investigated as follows: 600 μL of Fe^3+^ standard solution was added into 2.4 mL of PBS (20 mmol L^−1^, pH 7.02) aqueous solution. Then, 300 μL of Fe_3_O_4_@ZnO@L-Cys stocking solution was added into above solution and kept stewing for 30 min. Then, a magnet was used to separate the Fe^3+^-bound nanoprobes from aqueous solution. The assay method of the maximum adsorption amount of Fe_3_O_4_@ZnO@L-Cys toward Fe^3+^ was shown in supplementary materials.

### Application of Fe_3_O_4_@ZnO@L-Cys in real samples

Fresh human blood sample was obtained from the local hospital and pretreated according to the early published procedures^38^,^39^. In addition, the wastewater sample was collected from the local lake. The amount of Fe^3+^ was estimated using a standard addition method. For recovery studies, known concentrations of Fe^3+^ solution were added to the samples and the total iron concentrations were then determined at the same condition.

## Results and discussion

### Characterization of Fe_3_O_4_@ZnO@L-Cys

The morphology of Fe_3_O_4_ and Fe_3_O_4_@ZnO was observed by TEM. Fig. 2B showed the morphology of Fe_3_O_4_@ZnO. Compared with the bare Fe_3_O_4_ (Fig. 2A), it can be seen that ZnO was coated on the surface of Fe_3_O_4_ as a thin layer or single nanoparticle. Signal peaks for Fe, O and Zn were observed from the EDS spectrum (Fig. 2C) of Fe_3_O_4_@ZnO, indicating the successful synthesis of Fe_3_O_4_@ZnO. The FT-IR spectra of Fe_3_O_4_@ZnO@L-Cys were examined and shown in Fig. 2D. As shown, the peak at 1550–1650 cm^−1^ was corresponding to the C=O bending band. The bands located in the range of 600–800 cm^−1^ can be assigned to the C-S stretching vibration. The absorption band for the N-H was at 2900–3420 cm^−1^. The peak of 2550–2650 cm^−1^ which was related to the S-H for L-Cys^40^ disappears, indicating that the sulfur atom in mercapto group of L-Cys is coordinated with Zn^2+^ ions on the surface of the Fe_3_O_4_@ZnO.

### The interaction between Fe^3+^ and Fe_3_O_4_@ZnO@L-Cys

**Figure 1:**
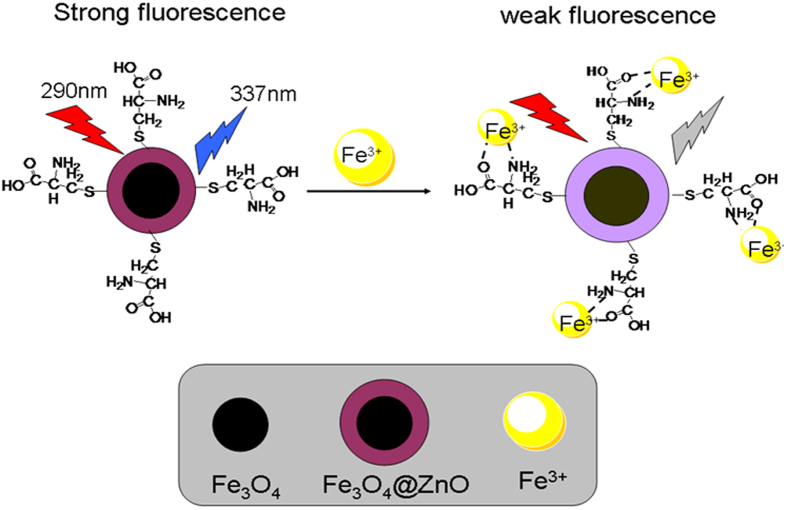
Structure of Fe_3_O_4_@ZnO@L-Cys and proposed binding mechanism of Fe^3+^ with Fe_3_O_4_@ZnO@L-Cys.

**Figure 2:**
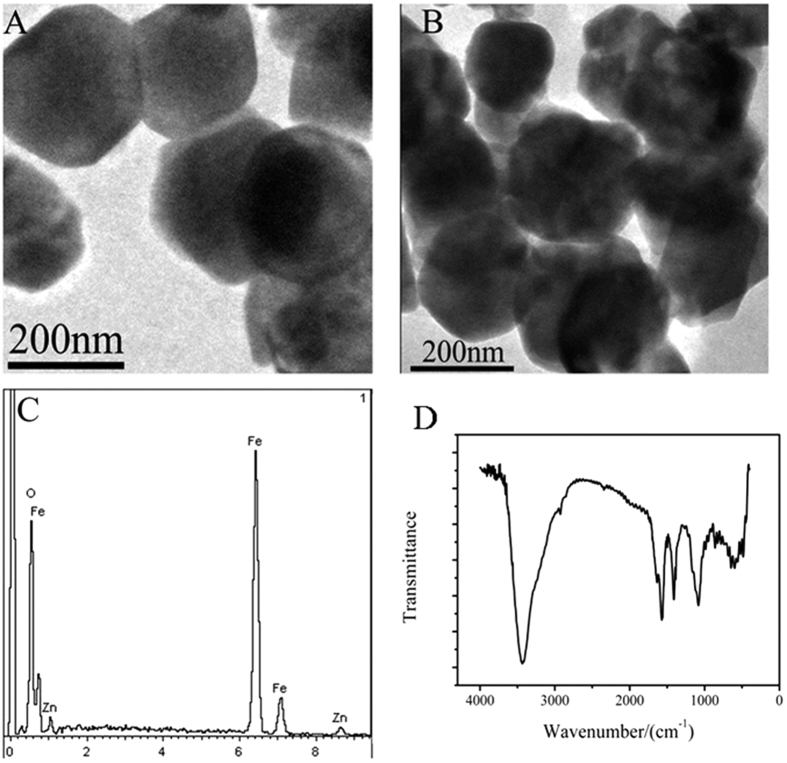
TEM images of Fe_3_O_4_ (**A**) and Fe_3_O_4_@ZnO (**B**); the EDS spectrum of Fe_3_O_4_@ZnO (**C**); IR spectra of Fe_3_O_4_@ZnO@L-Cys (**D**).

The absorption spectra of Fe_3_O_4_@ZnO in the presence of varying Fe^3+^ concentrations were investigated. As shown in Fig. S1, the main absorption band at approximately 380 nm of the Fe_3_O_4_@ZnO had a minor enhancement in the presence of 100 μmol L^−1^ Fe^3+^ without an obvious change of the peak shape. The slight changes of absorption spectra suggested that the quencher-Fe^3+^ did not affect the structure of the nanoparticles. The absorption band of Fe_3_O_4_@ZnO is usually very sensitive to the presence of adsorbed substances^41^,^42^. However, the presence of Fe^3+^ only generated slight changes in absorption spectra of the Fe_3_O_4_@ZnO@L-Cys. Thus, we may rule out the possibility of direct binding of Fe^3+^ to the Fe_3_O_4_@ZnO from the absorption spectra point of view. It could be clearly seen that the fluorescence intensity of the Fe_3_O_4_@ZnO@L-Cys was quenched dramatically with increase of Fe^3+^. So we speculated the added Fe^3+^ should interact with the L-Cys. Fe^3+^ ion is a well-known efficient fluorescence quencher due to its paramagnetic properties via electron or energy transfer. And L-cysteine, a common amino acid, possesses both amino and carboxyl function groups. It could be used to recognize the Fe^3+^ because the Fe^3+^ was known to be preferentially binding with nitrogen atom of imino group and oxygen atom of carbonyl group^20^,^43^. Thus we inferred the nitrogen atom of imino group and oxygen atom of carbonyl group in the L-Cys molecule might donor electrons to the Fe^3+^, as described in Fig. 1. In the same time, other interaction sites of six-coordinated Fe^3+^ may be occupied by the other Fe_3_O_4_@ZnO@L-Cys. Thus the coordination interaction occurred and induced intra-particles cross links which resulted in the fluorescence quenching^44^.

### Effect of pH values and ionic strength

**Figure 3:**
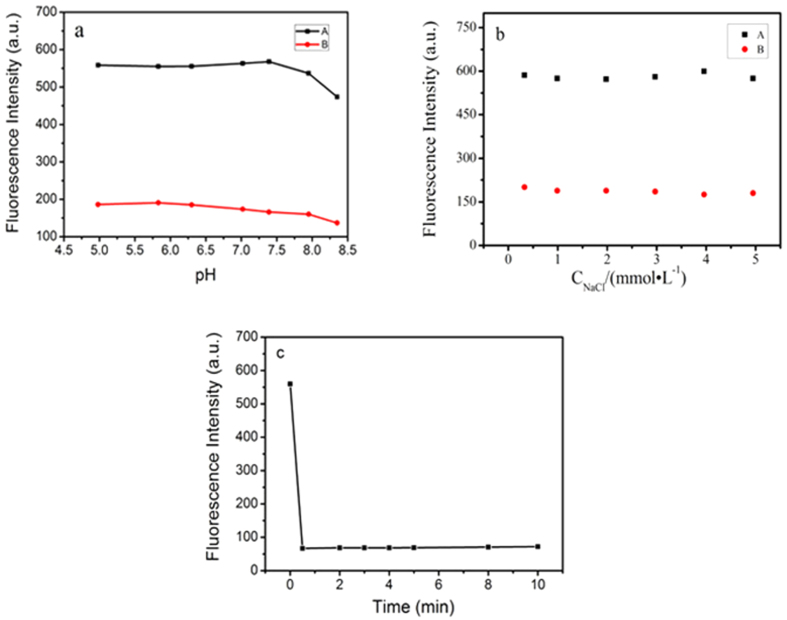
(**a**) Fluorescence intensity of our proposed nanosensor in the absence (A) and presence (B) of Fe^3+^ at different pH; (**b**) The effect of ionic strength on fluorescence intensity in the absence (A) and presence (B) of Fe^3+^; (**c**) Time course of the fluorescence response of Fe_3_O_4_@ZnO@L-Cys in the presence of Fe^3+^ (200 μmol L^−1^). The fluorescence intensity was recorded at 337 nm, with an excitation at 290 nm at room temperature.

Usually, the pH values of probes’ solution have tremendous influence on the detection of target analytes. So, the Fe^3+^-sensing ability of Fe_3_O_4_@ZnO@L-Cys at different pH was also investigated. The result showed that Fe_3_O_4_@ZnO@L-Cys was stable within a pH range from 4.98 to 7.39, and its response ability toward Fe^3+^ was stable within a pH range from 4.98 to 7.39 (Fig. 3a). Therefore, we choose the neutral aqueous solution (pH 7.02) as the analytical condition for the detection and removal of Fe^3+^.

The ionic strength was also a parameter for the detection of target analytes. The effect of ionic strength was presented in the Fig. 3b. As can be seen from the figure, the fluorescence intensity at 337 nm was not changed obviously before (Fig. 3b) and after (Fig. 3b) the addition of Fe^3+^ with the increasing concentration of NaCl solution, indicating the stability of the analytical platform at different ionic strength.

### Time course of the Fe_3_O_4_@ZnO@L-Cys toward Fe^3+^

Fig. 3c presents the response time of Fe_3_O_4_@ZnO@L-Cys toward Fe^3+^. As can be seen, the fluorescence intensity decreased rapidly within 1 min. At first the fluorescence intensity decreased minimum and then achieved a platform. Therefore, the fluorescent probe could realize the rapid analysis of Fe^3+^ in the samples.

### Determination of the standard solution of Fe^3+^

**Figure 4:**
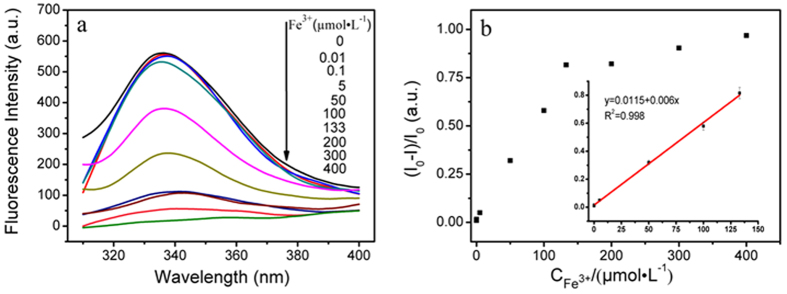
(**a**) Emission spectra of Fe_3_O_4_@ZnO@L-Cys in the presence of increasing amounts of Fe^3+^ at room temperature; (**b**) The curve of fluorescence intensity at 337 nm vs. Fe^3+^.

Quantitative detection of Fe^3+^ was carried out under PBS (20 mmol L^−1^, pH 7.02) aqueous solution. As shown in Fig. 4a, with the increasing concentration of Fe^3+^ (0, 0.01, 0.1, 5, 50, 100, 133, 200, 300, 400 μmol L^−1^), fluorescence intensity of Fe_3_O_4_@ZnO@L-Cys was decreased gradually and when the concentration of Fe^3+^ was 400 μmol L^−1^, the fluorescence of Fe_3_O_4_@ZnO@L-Cys was almost quenched. Furthermore, there was a linear relation between the relative fluorescence intensity at 337 nm and the concentration of Fe^3+^ varying from 0.01 to 133 μmol L^−1^ with a detection limit of 3 nmol L^−1^ (Fig. 4b). Compared with other reports (Table S1), the method we proposed can realize the real-time analysis of trace amount of Fe^3+^ with sensitivity and celerity. This may be attributed to the amount of amino and carboxyl groups on the surface of Fe_3_O_4_@ZnO.

### Selectivity and stability

**Figure 5:**
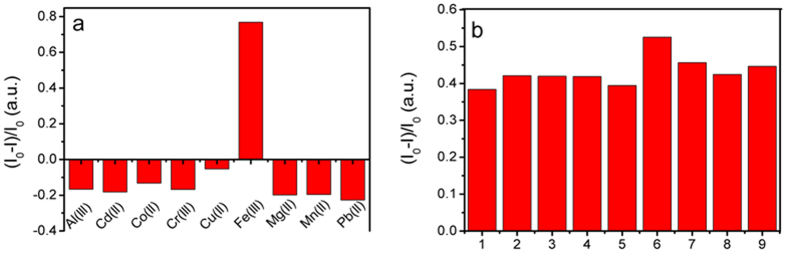
(**a**) The ratio of fluorescence quenching of Fe_3_O_4_@ZnO@L-Cys in the presence of different metal ions (200 μmol L^−1^); (**b**) The ratio of fluorescence quenching of Fe_3_O_4_@ZnO@L-Cys upon the addition of 1 equiv of Fe^3+^ to the solution containing 4 equiv of other metal ions (1, none; 2, Pb^2+^; 3, Al^3+^; 4, Mg^2+^; 5, Mn^2+^; 6, Cu^2+^; 7, Co^2+^; 8, Cr^3+^; 9, Cd^2+^).

High selectivity is a matter of necessity for an excellent sensor. Therefore, the selectivity of Fe_3_O_4_@ZnO@L-Cys for Fe^3+^ (200 μmol L^−1^) was investigated by screening its response to relevant analytes under the same condition. The results showed that other metal ions could enhance the fluorescence intensity of Fe_3_O_4_@ZnO@L-Cys, and the Fe^3+^ could decrease the fluorescence intensity of Fe_3_O_4_@ZnO@L-Cys (Fig. 5a). To further demonstrate the ability to recognize Fe^3+^ in the presence of other competitive mental ions (Al^3+^, Pb^2+^, Cr^3+^, Cd^2+^, Mg^2+^, Mn^2+^, Cu^2+^ and Co^2+^), the anti-interferential capability of the nanoparticle was also studied. When one equivalent of Fe^3+^ was added into the solution of the nanoparticle in the presence of four equivalents of other metal ions, higher concentration of the other metal ions did not affect the selectivity of Fe_3_O_4_@ZnO@L-Cys toward Fe^3+^ (Fig. 5b), except Cu^2+^ ion. This was because L-Cys molecule contained amino, carboxylic and thiol groups and many researches reported that the Cu^2+^ could bind with L-Cys^41^,^45^,^46^. Therefore, the Cu^2+^ showed an influence on the detection of Fe^3+^.

The stability of Fe_3_O_4_@ZnO@L-Cys was also examined. The fluorescence intensity of Fe_3_O_4_@ZnO@L-Cys at 337 nm was tested. After 20 d, the fluorescence intensity decreased to about 98% of its initial value, indicating the stability of Fe_3_O_4_@ZnO@L-Cys.

### Removal of Fe^3+^ from the standard solution

To investigate the removal ability of Fe_3_O_4_@ZnO@L-Cys, Fe^3+^ standard solution (3 mL, 400 μmol L^−1^) was chosen as testing solution. As indicated by Fig. S2A, the solution presented light yellow before the Fe_3_O_4_-based fluorescent nanoparticle was added into the solution. Then, 300 μL Fe_3_O_4_@ZnO@L-Cys stocking solution was added. A magnet was used to separate the Fe^3+^-bound nanosensors from aqueous solution after half an hour, the solution became clear and colorless (Fig. S2B), which indicated the Fe_3_O_4_@ZnO@L-Cys could be used for the extraction of Fe^3+^ from solution. Hence, the maximum adsorption amount of Fe3O4@ZnO@L-Cys toward Fe^3+^ was determined. And the result obtained by calculation is 192.64 mg/g, which can be seen clearly in Fig. S4.

### Determination of iron contents in real samples

The serum and the wastewater sample were determined and the results were shown in Table S2. The determinated iron contents were at reasonable range in according to the literature values detected with other approaches, such as the methods of fluorescent gold nanoclusters^38^, atomic absorption spectrometry^47^ and inductively coupled plasma mass spectrometry^48^. The recoveries of the known amount Fe^3+^ in serum samples were 92.6–108.4%, while in wastewater samples were 89.6–113.0%. The results demonstrated reliability of Fe_3_O_4_@ZnO@L-Cys for detecting iron contents in real samples.

## Conclusion

In summary, a really facile detection method based on fluorescent probe Fe_3_O_4_@ZnO@L-Cys has been developed, which allowed the highly sensitive and selective determination of Fe^3+^. It is the first time to apply Fe_3_O_4_@ZnO based sensing platform for the analysis of iron contents. And the magnetic nanoparticle Fe_3_O_4_@ZnO could be prepared easily and environmental friendly. The fluorescence intensity of fluorescent probe Fe_3_O_4_@ZnO@L-Cys was quenched significantly in the presence of Fe^3+^ within 1 min. Other common metal ions at four times concentrations of Fe^3+^ did not cause interference. Furthermore, the proposed fluorescent probe could be applied to detect iron contents in real samples and extract the Fe^3+^ from the solution which containing high concentration of Fe^3+^ with the aid of external magnetic field.

## Additional Information

**How to cite this article**: Li, J. *et al*. Highly selective fluorescent chemosensor for detection of Fe^3+^ based on Fe_3_O_4_@ZnO. *Sci. Rep.* **6**, 23558; doi: 10.1038/srep23558 (2016).

## Supplementary Material

Supplementary Information
