# Supplementary material for: Highly selective fluorescent chemosensor for detection of Fe3+ based on Fe3O4@ZnO
Source: Sci Rep. 2016 Mar 22;6:23558. doi: 10.1038/srep23558 (PMC4802302; doi:10.1038/srep23558)
Supplement: Supplementary Information [file srep23558-s1.doc]

*Supporting Information*

**Highly selective fluorescent chemosensor for detection of Fe^3+^ based on Fe_3_O_4_@ZnO**

Jingshuai Li^a^, Qi Wang^b^, Zhankui Guo^a^, Hongmin Ma^a^, Yong Zhang^a^, Bing Wang^a^, Bin Du^a^, Qin Wei*^a^

^a^Key Laboratory of Chemical Sensing & Analysis in Universities of Shandong, School of Chemistry and Chemical Engineering, University of Jinan, Jinan 250022, P.R. China

^b^School of Material Science and Engineering, University of Jinan, Jinan 250022, P.R. China

Correspondence: [sdjndxwq@163.com](mailto:sdjndxwq@163.com)

Tel: (86) 531-82767872

Fax: (86) 531-82767367

**The maximum adsorption amount of Fe_3_O_4_@ZnO@L-Cys**

The determination of the standard curve of iron ions was carried out according to the following steps: different volumes of Fe^3+^ stocking solution was added into 1.0 mL of HAc-NaAc (pH 4.00) aqueous solution respectively. Then 600 µL of sodium sulfosalicylate was added and the total volume of the solution is 10 mL. After a period of time, the ultraviolet absorption spectra of different concentrations of Fe^3+^ (0.5, 5, 10, 30, 50, 100, 150, 200, 300, 400 µmol L^-1^) were measured. In order to study the adsorption capacity of magnetic fluorescent probes that we prepared, 440 µg of the probe was dispersed into 5.0 mL of PBS (20 mmol L^-1^, pH 7.02) aqueous solution. Then different concentrations of Fe^3+^ (5, 20, 50, 100, 150, 200, 300, 400 µmol L^-1^) were added and the final volume of the solution is 10 mL. A magnet was used to separate the Fe^3+^-bound nanoprobes from aqueous solution and then 8 mL of the supernatant was removed. The concentration of iron ions in the solution was measured by ultraviolet absorption spectra.

Quantification of Fe^3+^ was carried out under HAc-NaAc (pH 4.00) aqueous solution. As shown in Fig. S3A, with the increasing concentration of Fe^3+^, the absorbance increased gradually. Furthermore, there was a linear relation between the absorbance at 464 nm and the concentration of Fe^3+^ (Fig. S3B). The adsorption ability of magnetic fluorescent probes is shown in Fig. S4. The probe’ adsorption capacity of per gram is calculated by the following formula: $Q=\left[\left(C_{0}-C\right)\times V\right]\times m^{-1}$. $C_{0}$ and $C$ are the initial and final concentration of iron ions in the solution, respectively; $V$ is the volume of the sample; $m$ is the quality of the probe. The maximum adsorption amount of Fe_3_O_4_@ZnO@L-Cys toward Fe^3+^ obtained by calculation is 192.64 mg/g. The results show that Fe_3_O_4_@ZnO@L-Cys possesses excellent adsorption properties.

Fig. S5 showed the UV-vis absorption spectra of Fe_3_O_4_@ZnO (a), L-Cys (b) and Fe_3_O_4_@ZnO@L-Cys (c). From the absorption spectra, it is observed that pure L-Cys shows an absorption peak at 255 nm. With the formation of Fe_3_O_4_@ZnO@L-Cys, the intensity of the characteristic peak of L-Cys is red shifted as observed, which could attributed to the interaction between Fe_3_O_4_@ZnO and L-Cys. Additionally, it could be observed that the Fe_3_O_4_@ZnO@L-Cys exhibited a sharp absorption peak at 222 nm.


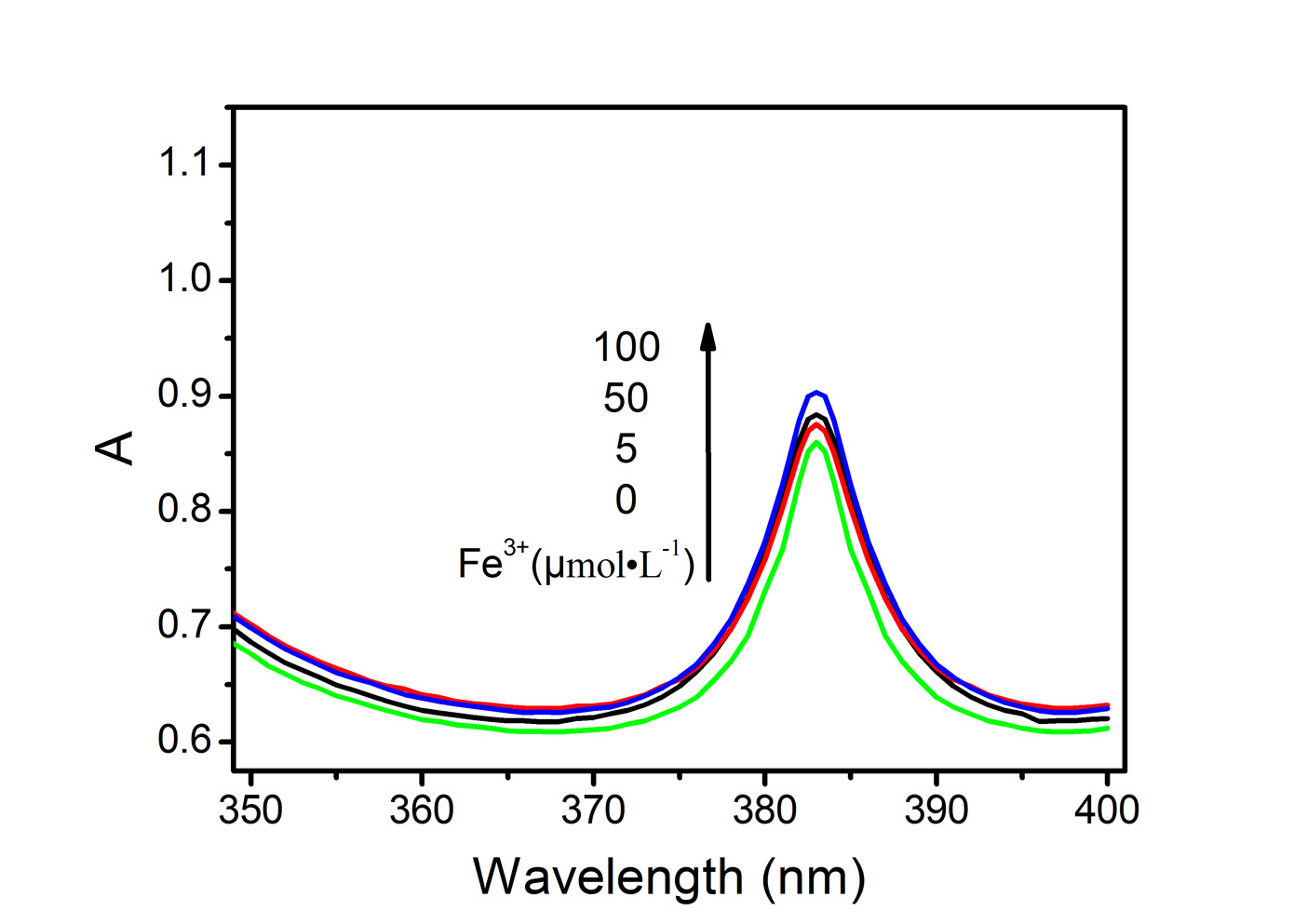


**Figure S1.** Absorption spectra of Fe_3_O_4_@ZnO@L-Cys in the presence of varying concentrations of Fe^3+^ (0, 5, 50, 100 µmol L^-1^).


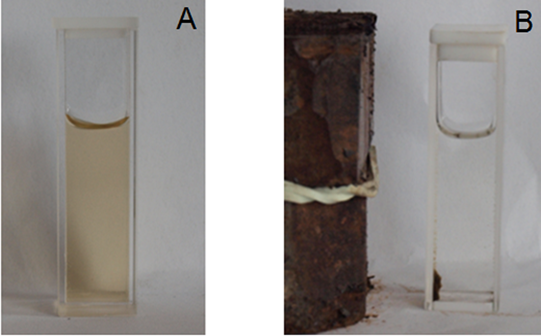


**Figure S2.** (A) Photograph of Fe^3+^ solution (400 µmol L^-1^); (B) Photograph of an external magnet separating the proposed nanoparticle for the removal of Fe^3+^.

**
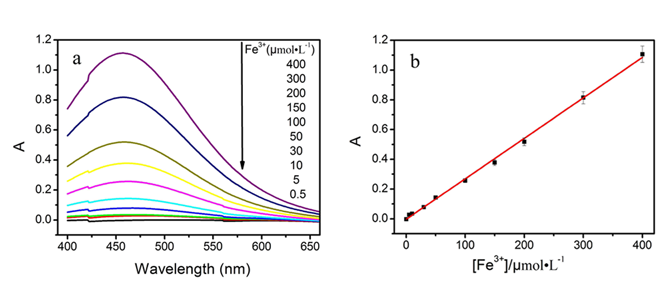
**

**Figure S3.** (a) Ultraviolet absorption spectra of different concentrations of Fe^3+^; (b) The curve of absorbance at 464 nm vs. Fe^3+^.


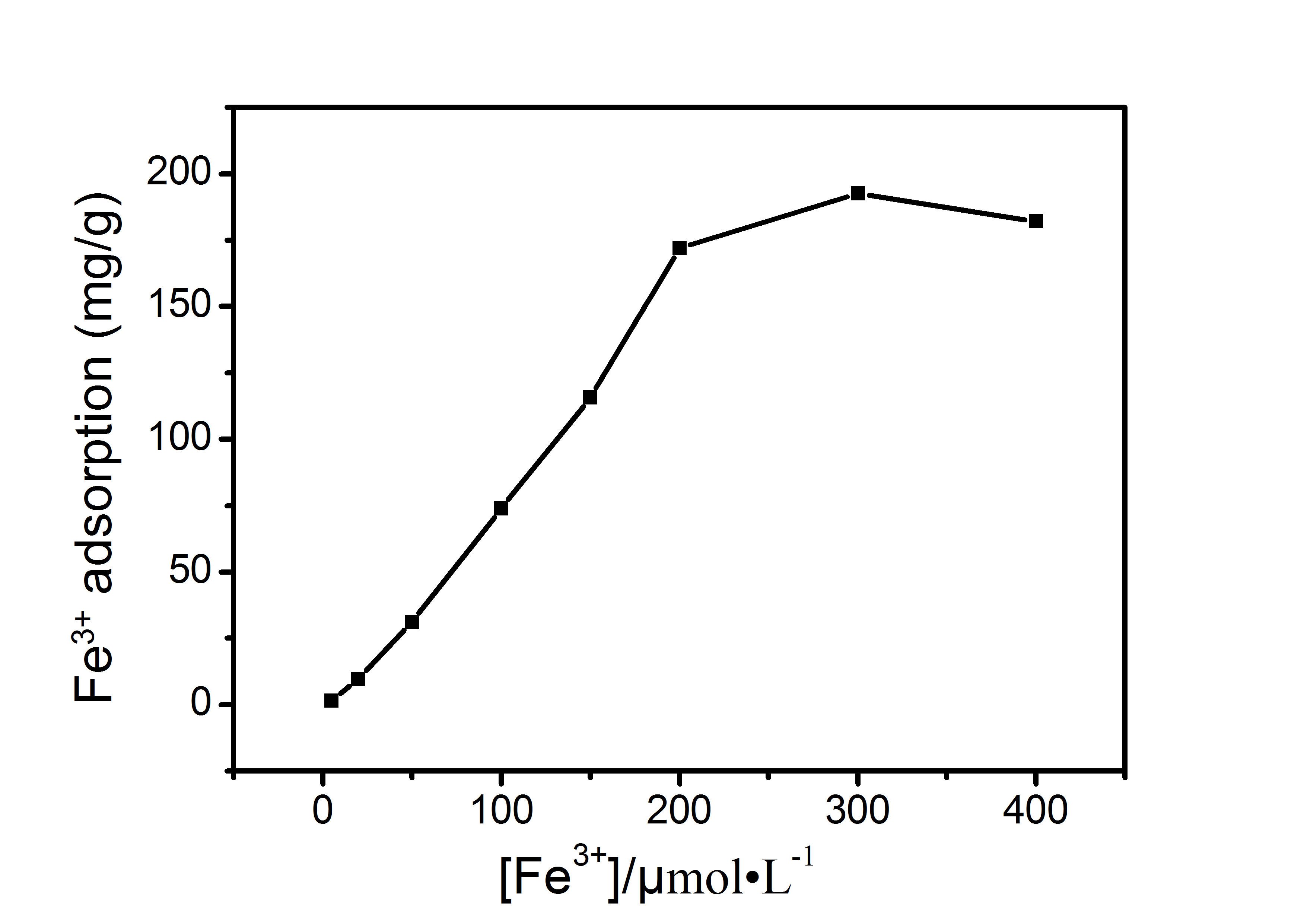


**Figure S4.** Adsorption ability of the probe with increasing amounts of Fe^3+^ (5-400 µmol L^-1^).

**
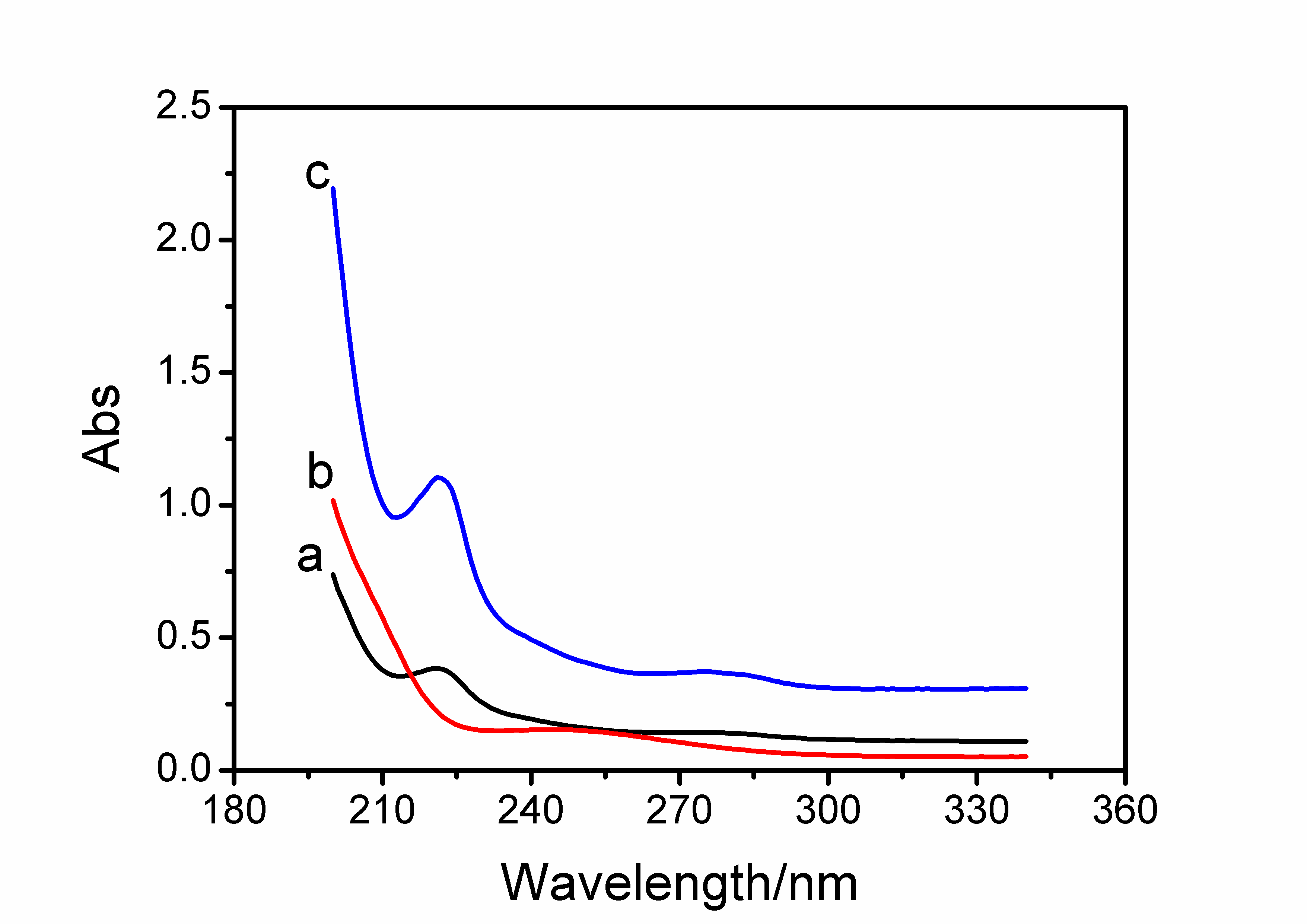
**

**Figure S5.** UV-vis absorption spectra of Fe_3_O_4_@ZnO (a), L-Cys (b) and Fe_3_O_4_@ZnO@L-Cys (c).

**Table S1 Comparison with other methods in the detection of Fe^3+^**

| Methods | Speed | Dynamic range  (µmol·L^-1^) | Detection limit  (µmol·L^-1^) | References |
| --- | --- | --- | --- | --- |
| Fluorescence | 600 s | 0.5 ~ 20 | 0.12 | [^1^](#_ENREF_1) |
| Fluorescence | 20 s | 0.5 ~ 9 | 0.01 | [^2^](#_ENREF_2) |
| Fluorescence ratiometric | 60 s | 0 ~ 20 | 0.105 | [^3^](#_ENREF_3) |
| Colorimetric | -- | 0.5 ~ 80 | 1.79 | [^4^](#_ENREF_4) |
| Electrochemical | -- | 0.30 ~ 100 | 0.035 | [^5^](#_ENREF_5) |
| Atomic absorption | -- | 0.05 ~ 1.5 | 0.006 | [^6^](#_ENREF_6) |
| Fluorescence | 30 s | 0.01 ~ 400 | 0.003 | This work |

**Table S2 Determination of iron contents in real samples**

| Real  Samples | Determined Fe^3+^  (10^−6^ mol L^-1^) | Added  (10^−6^ mol L^-1^) | Found  (10^−6^ mol L^-1^) | Recovery (%) | RSD  (*n*=5, %) |
| --- | --- | --- | --- | --- | --- |
| Human serum | 5.16 | 2.00 | 6.98 | 92.6 | 4.1 |
|  |  | 5.00 | 10.54 | 108.4 | 3.4 |
|  |  | 8.00 | 12.95 | 98.8 | 6.5 |
| Wastewater | 0.25 | 1.00 | 1.23 | 113.0 | 5.3 |
|  |  | 3.00 | 3.26 | 102.0 | 8.1 |
|  |  | 5.00 | 4.65 | 89.6 | 6.8 |

**References**

1. Chen, Z. et al. Glutathione capped silver nanoclusters-based fluorescent probe for highly sensitive detection of Fe^3+^. *Sensor. Actuat. B-Chem.* **202**, 631-637 (2014).

2. Zhang, Y., Wang, G. & Zhang, J. Study on a highly selective fluorescent chemosensor for Fe^3+^ based on 1, 3, 4-oxadiazole and phosphonic acid. *Sensor. Actuat. B-Chem.* **200**, 259-268 (2014).

3. Wang, C., Huang, X., Ding, P., Wang, Z., Zhao, Y. & Ye, Y. A fluorescence ratiometric chemosensor for Fe^3+^ based on TBET and its application in living cells. *Talanta.* **128**, 69-74 (2014).

4. Devaraj, S., Tsui, Y.K., Chiang, C.Y. & Yen, Y.P. A new dual functional sensor: highly selective colorimetric chemosensor for Fe^3+^ and fluorescent sensor for Mg^2+^. *Spectrochim. Acta. A.* **96**, 594-599 (2012).

5. Li, F. et al. Electrochemical determination of iron in coastal waters based on ionic liquid-reduced graphene oxide supported gold nanodendrites. *Electrochim. Acta.* **176**, 548-554 (2015).

6. Hosseini, M.S., Raissi, H. & Madarshahian, S. Synthesis and application of a new chelating resin functionalized with 2,3-dihydroxy benzoic acid for Fe(III) determination in water samples by flame atomic absorption spectrometry. *React. Funct. Polym.* **66**, 1539-1545 (2006).
